# In Memoriam

**Published:** 1893-11

**Authors:** 


### IN MEMORIAM—DR. SAMUEL SWAN.

The homœopathic profession will read with surprise this announcement of the death of Dr. Samuel Swan, of New York, so widely known in connection with the numerous remedies of the nosode class which he has from time to time introduced to professional notice. He died on Wednesday the 18th of October, at ten o'clock in the morning. Dr. Swan had been unwell for several months and had relaxed his usual active work and had lost much of his interest in the events of the profession owing to the prostration that his illness had caused. In May last he wrote: "I have been sick for the last two months and am now so weak I can't attend to patients, who very considerately keep away from me. * * * I have been too sick to pay attention to anything." Later he retired to his bed never to rise again. He was well aware his end was approaching and expressed a wish to die. He was fully conscious to the last, but had lost the use of his voice and was finally unable through weakness even to write what he wished to say.

Dr. Swan was born at Medford, Mass., July 4th, 1815. He was therefore seventy-eight years old at the time of his death.

The first half of his life was spent in active business pursuits, which he was finally compelled to relinquish on account of ill health. He then went South in the company of his devoted wife, and there met Dr. Uhlrick through whom he became interested in medicine, and under whose direction he studied it. He then returned to the North, entered as a student in the Homœopathic Medical College of Pennsylvania, at Philadelphia, now merged in Hahnemann Medical College, and graduated in 1866. His diploma bears the signatures of such distinguished men as Adolph Lippe, Constantine Hering, and Henry N. Guernsey. He settled in New York, and went into the active practice of medicine, which he continued until incapacitated by the illness of the last few months. The first five years of his medical career were spent almost entirely in gratuitous practice. He was a kind man and none who appealed to him for aid ever went away unsatisfied. The grateful memories that cluster about his name amply testify to his deeds of good-will and benevolence.

The writer of this sketch himself owes Dr. Swan a large debt of gratitude for valuable professional services rendered to his mother under the following circumstances:

The patient had been suffering for years the most excruciating agony from headache. The pain was so violent as to cause loud screaming and a desire to run wildly from one room to another. No remedies prescribed seemed to have any effect, and there seemed to be no hope of procuring relief.

In June of 1876, Dr. Adolph Lippe gave a dinner party at which were assembled Dr. Edward Bayard, Dr. Henry N. Guernsey, Dr. Constantine Lippe, Dr. Samuel Swan, two or three others whose names it is impossible now to recall, and the writer. This case of violent headache was incidentally mentioned to Dr. Swan in the course of a conversation in which experiences had been mutually recounted.

He became much interested and offered to prescribe. A detailed statement of the symptomatology was furnished him, and after two or three remedies had been given with but indifferent success, *Lac-felinum* was administered. The screaming ceased and the headache slowly disappeared. The disease energy was driven to the surface with the production of an extremely annoying eruption upon the legs of a decidedly erysipelatous character which has continued from that time. The relief from the intense agony of the headache, however, was as complete as it was remarkable.

In January, 1878, he joined Dr. Thomas Skinner, then of Liverpool, and now of London, Dr. Adolph Lippe, of Philadelphia, and Dr. Berridge, of London, in the publication of a new journal devoted to pure Homœopathy. It was called *The Organon*, and was issued quarterly. It at once took a prominent position in medical journalism, and promised to be a great success. It ceased after three years of publication, however, and pure Homœopathy was without a representative. It was then that Dr. Lippe, deploring the loss of the journal, determined to start another journal in its place. THE HOMŒOPATHIC PHYSICIAN was thus established and was the successor of *The Organon.* Dr. Swan became much interested in this latest venture and was a frequent contributor to its pages.

Dr. Swan did not confine himself to pure Homœopathy, and he soon became widely known for his indorsement of Isopathy. This was considered to be an invasion, and a nullification of the doctrine of the law of similars, and it brought upon him a storm of denunciations and criticisms in which this journal sometimes participated. It would be out of place here to rekindle the fires of that controversy, but without affirming or denying the injurious effect upon Homœopathy that it is claimed to have caused, the one practical result has been the bringing to professional notice of a large number of new and singular remedies. Among these may be mentioned the various "milks" which were, with one exception, introduced by Dr. Swan. That one exception was Lac-caninum, which was originated by Dr. Reissig; by him communicated to Dr. Bayard, who, in turn, transmitted the information to Dr. Swan.

Dr. Swan introduced Tuberculinum to medicine years before the same remedy was discovered by Professor Koch, of Berlin, who made such a tremendous sensation with it in the ranks of the dominant school. He also introduced Syphilinum, Medorrhinum, and other remedies of like character, now known under the general name of nosodes.

The profession were not opposed to the use of these nosodes, but the demand was frequently made that they be proved like the "polycrests." To this Dr. Swan answered that these remedies had already produced provings which could be found in the phenomena and symptoms of the disease of which they were the products. This answer did not satisfy the strictly logical Hahnemannians, and thus a gulf was formed between them and him which has continually widened. Much more might be said, but space will not permit the elaboration of the subject, and it is accordingly left to other writers to treat as they shall feel inspired.

